# Supplementary material for: A Global Study on the Correlates of Gross Domestic Product (GDP) and COVID-19 Vaccine Distribution
Source: Vaccines (Basel). 2022 Feb 10;10(2):266. doi: 10.3390/vaccines10020266 (PMC8879784; doi:10.3390/vaccines10020266)
Supplement: Supplementary file 1 [file vaccines-10-00266-s001.zip › vaccines-1557202-supplementary.pdf]

Table S1. R-Studio Project files including R-code and raw data used in the analysis

Country	Continent	GDP per capita	People Vaccinated (%)
Afghanistan	Asia	1803.987	11.04
Albania	Europe	11803.431	39.39
Algeria	Africa	13913.839	15.73
Angola	Africa	5819.495	21.88
Antigua and Barbuda	North America	21490.943	62.95
Argentina	South America	18933.907	82.98
Armenia	Asia	8787.58	30.45
Aruba	North America	35973.781	78.84
Australia	Oceania	44648.71	79.09
Austria	Europe	45436.686	72.84
Azerbaijan	Asia	15847.419	50.41
Bahamas	North America	27717.847	39.23
Bahrain	Asia	43290.705	68.57
Bangladesh	Asia	3523.984	52.64
Barbados	North America	16978.068	54.03
Belarus	Europe	17167.967	46.13
Belgium	Europe	42658.576	76.51
Belize	North America	7824.362	56.73
Benin	Africa	2064.236	12.54
Bermuda	North America	50669.315	74.4
Bhutan	Asia	8708.597	75.62
Bolivia	South America	6885.829	46.06
Bosnia and Herzegovina	Europe	11713.895	25.53
Botswana	Africa	15807.374	47.21
Brazil	South America	14103.452	77.52
Brunei	Asia	71809.251	91.75
Bulgaria	Europe	18563.307	15.79
Burkina Faso	Africa	1703.102	4.52
Burundi	Africa	702.225	0.03
Cambodia	Asia	3645.07	84.03
Cameroon	Africa	3364.926	2.96
Canada	North America	44017.591	83.11
Cape Verde	Africa	6222.554	53.89
Cayman Islands	North America	49903.029	88.08
Central African Republic	Africa	661.24	9.08
Chad	Africa	1768.153	1.69
Chile	South America	22767.037	89.55
China	Asia	15308.712	84.82
Colombia	South America	13254.949	74.26
Comoros	Africa	1413.89	31.78
Congo	Africa	4881.406	12.99
Costa Rica	North America	15524.995	76.57
Cote d'Ivoire	Africa	3601.006	12.22
Croatia	Europe	22669.797	55.15
Cyprus	Europe	32415.132	71.53
Czechia	Europe	32605.906	63.41
Democratic Republic of Congo	Africa	808.133	0.24
Denmark	Europe	46682.515	82.29
Djibouti	Africa	2705.406	10.87

Dominica	North America	9673.367	40.77
Dominican Republic	North America	14600.861	63.29
Ecuador	South America	10581.936	78.49
Egypt	Africa	10550.206	30.79
El Salvador	North America	7292.458	68.68
Equatorial Guinea	Africa	22604.873	17.19
Estonia	Europe	29481.252	63.35
Eswatini	Africa	7738.975	28.38
Ethiopia	Africa	1729.927	7.89
Fiji	Oceania	8702.975	73.26
Finland	Europe	40585.721	78.3
France	Europe	38605.671	77.98
Gabon	Africa	16562.413	17.24
Gambia	Africa	1561.767	9.81
Georgia	Asia	9745.079	33.43
Germany	Europe	45229.245	73.19
Ghana	Africa	4227.63	18.2
Greece	Europe	24574.382	71.46
Grenada	North America	13593.877	36.33
Guatemala	North America	7423.808	35.47
Guinea	Africa	1998.926	12.89
Guinea-Bissau	Africa	1548.675	19.33
Guyana	South America	7435.047	55.68
Haiti	North America	1653.173	1.06
Honduras	North America	4541.795	49.1
Hong Kong	Asia	56054.92	64.23
Hungary	Europe	26777.561	64.83
Iceland	Europe	46482.958	83.82
India	Asia	6426.674	59.91
Indonesia	Asia	11188.744	56.24
Iran	Asia	19082.62	69.54
Iraq	Asia	15663.986	20.38
Ireland	Europe	67335.293	78.02
Israel	Asia	33132.32	69.94
Italy	Europe	35220.084	79.68
Jamaica	North America	8193.571	23.86
Japan	Asia	39002.223	79.54
Jordan	Asia	8337.49	41.89
Kazakhstan	Asia	24055.588	47.02
Kenya	Africa	2993.028	11.52
Kiribati	Oceania	1981.132	49.01
Kosovo	Europe	9795.834	49.49
Kuwait	Asia	65530.537	76.92
Kyrgyzstan	Asia	3393.474	18.3
Laos	Asia	6397.36	50.75
Latvia	Europe	25063.846	70.03
Lebanon	Asia	13367.565	32.46
Lesotho	Africa	2851.153	30.05
Liberia	Africa	752.788	15.15
Libya	Africa	17881.509	26.24

Lithuania	Europe	29524.265	70.98
Luxembourg	Europe	94277.965	16.31
Macao	Asia	104861.851	74.68
Madagascar	Africa	1416.44	2.07
Malawi	Africa	1095.042	7.28
Malaysia	Asia	26808.164	79.21
Maldives	Asia	15183.616	72.94
Mali	Africa	2014.306	4.15
Malta	Europe	36513.323	85.31
Mauritania	Africa	3597.633	23.41
Mauritius	Africa	20292.745	73.59
Mexico	North America	17336.469	62.88
Moldova	Europe	5189.972	13.94
Mongolia	Asia	11840.846	68.04
Montenegro	Europe	16409.288	44.97
Morocco	Africa	7485.013	65.9
Mozambique	Africa	1136.103	24.39
Myanmar	Asia	5591.597	34.36
Namibia	Africa	9541.808	15.45
Nauru	Oceania	12895.635	70.01
Nepal	Asia	2442.804	41.28
Netherlands	Europe	48472.545	72.93
New Zealand	Oceania	36085.843	77.52
Nicaragua	North America	5321.444	69.06
Niger	Africa	926	2.02
Nigeria	Africa	5338.454	4.37
North Macedonia	Europe	13111.214	40.35
Norway	Europe	64800.057	78.3
Oman	Asia	37960.709	59.76
Pakistan	Asia	5034.708	40.89
Palestine	Asia	4449.898	39.31
Panama	North America	22267.037	69.01
Papua New Guinea	Oceania	3823.194	3.11
Paraguay	South America	8827.01	47.71
Peru	South America	12236.706	72.2
Philippines	Asia	7599.188	50.53
Poland	Europe	27216.445	56.94
Portugal	Europe	27936.896	90.28
Qatar	Asia	116935.6	80.54
Romania	Europe	23313.199	28.64
Russia	Europe	24765.954	49.73
Rwanda	Africa	1854.211	55.16
Saint Kitts and Nevis	North America	24654.385	51.4
Saint Lucia	North America	12951.839	30.11
Saint Vincent and the Grenadines	North America	10727.146	29.94
Samoa	Oceania	6021.557	70.66
San Marino	Europe	56861.47	71.47
Sao Tome and Principe	Africa	3052.714	38.68
Saudi Arabia	Asia	49045.411	70.55
Senegal	Africa	2470.58	7.72

Serbia	Europe	14048.881	48.06
Seychelles	Africa	26382.287	83.23
Sierra Leone	Africa	1390.3	9.18
Singapore	Asia	85535.383	88
Sint Maarten (Dutch part)	North America	36327.232	62.38
Slovakia	Europe	30155.152	49.66
Slovenia	Europe	31400.84	60.01
Solomon Islands	Oceania	2205.923	25.56
South Africa	Africa	12294.876	46.27
South Korea	Asia	35938.374	85.54
South Sudan	Africa	1569.888	2
Spain	Europe	34272.36	84.3
Sri Lanka	Asia	11669.077	74.33
Sudan	Africa	4466.507	8.94
Suriname	South America	13767.119	44.1
Sweden	Europe	46949.283	76.15
Switzerland	Europe	57410.166	68.34
Tajikistan	Asia	2896.913	36.11
Tanzania	Africa	2683.304	2.59
Thailand	Asia	16277.671	72.58
Timor	Asia	6570.102	49.33
Togo	Africa	1429.813	16.12
Tonga	Oceania	5425.621	68.95
Trinidad and Tobago	North America	28763.071	50.4
Tunisia	Africa	10849.297	54.98
Turkey	Asia	25129.341	66.76
Turkmenistan	Asia	16389.023	71.51
Tuvalu	Oceania	3575.104	52.24
Uganda	Africa	1697.707	17.73
Ukraine	Europe	7894.393	33.53
United Arab Emirates	Asia	67293.483	98.99
United Kingdom	Europe	39753.244	75.72
United States	North America	54225.446	72.75
Uruguay	South America	20551.409	79.3
Uzbekistan	Asia	6253.104	38.13
Vanuatu	Oceania	2921.909	32.53
Venezuela	South America	16745.022	64.08
Vietnam	Asia	6171.884	76.04
Yemen	Asia	1479.147	1.83
Zambia	Africa	3689.251	4.26
Zimbabwe	Africa	1899.775	27.14

People Fully Vaccinated (%)	Population
8.95	39835428
35.45	2872934
12.45	44616626
11.03	33933611
59.26	98728
70.4	45605823
21.66	2968128
73.52	107195
76.31	25788217
70.77	9043072
45.54	10223344
37.17	396914
67.09	1748295
26.9	166303494
49.6	287708
34.19	9442867
75.55	11632334
48.95	404915
9.63	12451031
72.82	62092
72.46	779900
38.09	11832936
22.08	3263459
41.95	2397240
66.74	213993441
87.29	441532
15.76	6896655
3.02	21497097
0.03	12255429
80.35	16946446
2.3	27224262
77.11	38067913
45.55	561901
85.51	66498
6.99	4919987
0.48	16914985
85.64	19212362
74.53	1444216102
54.08	51265841
27.55	888456
9.91	5657017
67.02	5139053
5.57	27053629
51.9	4081657
67.2	896005
61.69	10724553
0.11	92377986
78.03	5813302
2.6	1002197

38.27	72172
51.94	10953714
67.88	17888474
18.78	104258327
63.61	6518500
14.03	1449891
61.36	1325188
25.55	1172369
1.24	117876226
67.07	902899
74.3	5548361
72.72	67564251
7.6	2278829
9.25	2486937
27.92	3979773
70.19	83900471
7.36	31732128
66.77	10370747
31.03	113015
25.13	18249868
6.22	13497237
1.13	2015490
39.78	790329
0.62	11541683
39.28	10062994
61.29	7552800
61.84	9634162
82.61	343360
40.89	1393409033
39.79	276361788
59.2	85028760
13.41	41179351
76.86	4982904
63.21	9291000
73.83	60367471
18.83	2973462
78.12	126050796
37.89	10269022
44.4	18994958
7.02	54985702
18.68	121388
43.71	1782115
74.16	4328553
14.87	6628347
42	7379358
67.09	1866934
26.57	6769151
29.69	2159067
14.58	5180208
11.39	6958538

67.78	2689862
5.97	634814
69.57	658391
1.9	28427333
3.39	19647681
78.02	32776195
67.46	543620
1.8	20855724
84.3	516100
15.09	4775110
71.36	1273428
55.77	130262220
13.14	4024025
64.94	3329282
42.96	628051
61.49	37344787
16.8	32163045
23.77	54806014
13.01	2587344
66.69	10873
32.81	29674920
67.04	17173094
74.77	5122600
39.98	6702379
1.85	25130810
2.01	211400704
38.97	2082661
71.59	5465629
55.34	5223376
28.93	225199929
28.31	5222756
56.17	4381583
2.41	9119005
39.77	7219641
62.39	33359415
33.64	111046910
55.35	37797000
89.31	10167923
75.7	2930524
27.91	19127772
44.61	145912022
37.19	13276517
47.44	53546
26.54	184401
23.11	111269
60.84	200144
63.58	34010
22.93	223364
65.19	35340680
5.47	17196308

46.47	6871547
79.1	98910
4.73	8141343
87	5453600
57.83	43421
44.19	5460726
57.05	2078723
7.95	703995
26.23	60041996
82.37	51305184
1.6	11381377
80.93	46745211
64.31	21497306
2.75	44909351
38.35	591798
72.31	10160159
66.64	8715494
28.38	9749625
1.75	61498438
64.07	69950844
39.66	1343875
10.95	8478242
53.04	106759
47.35	1403374
47.22	11935764
60.4	85042736
52.41	6117933
49.34	11925
2.99	47123533
30.82	43466822
90.68	9991083
69.28	68207114
61.19	332915074
76.66	3485152
17.33	33935765
16.03	314464
40.44	28704947
57.44	98168829
1.2	30490639
3.31	18920657
20.57	15092171
